# One-pot neutron imaging of surface phenomena, swelling and diffusion during methane absorption in ethanol and *n*-decane under high pressure

**DOI:** 10.1371/journal.pone.0238470

**Published:** 2020-09-10

**Authors:** Ondřej Vopička, Petr Číhal, Martina Klepić, Jan Crha, Vladimír Hynek, Karel Trtík, Pierre Boillat, Pavel Trtik

**Affiliations:** 1 Department of Physical Chemistry, University of Chemistry and Technology, Prague, Czech Republic; 2 Laboratory of Neutron Scattering and Imaging, Paul Scherrer Institut, Villigen, Switzerland; 3 Universita třetího věku (U3V), Czech Technical University in Prague, Prague, Czech Republic; University of New South Wales, AUSTRALIA

## Abstract

We report a powerful method for capturing the time-resolved concentration profiles, liquid swelling and surface phenomena during the absorption of methane (CH_4_) in still liquid ethanol (C_2_D_6_O) and *n*-decane (*n*-C_10_D_22_) and at high spatial resolution (pixel size 21.07 μm) using neutron imaging. Absorption of supercritical methane was followed at two temperatures and two pressures of methane, namely 7.0, 37.8 °C and 80, 120 bar. Fick’s second law, which was used in the liquid-fixed coordinates, enabled for an adequate parameterization of the observed concentration profiles and liquid levels using simple analytical expressions. For both studied liquids, anomalously slow diffusion was observed in the initial stage of the absorption experiment. This was ascribed to the slow formation of the surface excess on the interface, time constant ranged 130–275 s. The axial symmetry of the cell allowed for the tomographic reconstructions of the profiles of the menisci. Based on these profiles, contact angle and surface tension were evaluated using the Young-Laplace equation. Overall, neutron imaging made it possible to capture time- and space-resolved information from which the methane concentration, liquid level and meniscus shape under high-pressure conditions inside a cylindrical titanium vessel were quantitatively derived. Multiple characteristics of ethanol, a methane hydrate inhibitor, and *n*-decane, a model constituent of crude oil, were thus measured for the first time under industrially relevant conditions in a one-pot experiment.

## Introduction

Methane has been forecast to become the second most used energy resource within the next two decades [1–3]. The production of this gas, which can be seen as a bridge to low-carbon energy future [4], is inherently related to its basic properties, such as its ability to absorb and diffuse in liquids. Methane absorption naturally influences the properties of the liquid, such as density and surface tension.

Ethanol is an industrially relevant inhibitor of the methane hydrate formation [5], for which equilibrium methane solubility and the density of the solution under high pressure were reported in the literature [6–8]. *n*-decane is a model constituent of crude oil. Literature data on the methane solubility and diffusivity and on surface tension are available either for *n*-decane and methane or for similar systems under high pressures [9–18]. Clearly, mixtures of a light gas with a heavy solvent are relevant for the production and processing of crude oil and natural gas [19, 20].

The classical methods for the measurement of the transient absorption of gases in liquid bodies are indirect: the amount of gas dissolved in the liquid is measured [10, 11, 21], while the true distribution of the compounds remains the subject of assumptions. Diffusion in diluted systems under high pressure can also be measured using the Taylor dispersion method [22]. Once equilibrium is reached, the equilibrium solubility and the liquid density can be measured by several well established methods [7, 8, 23]. Besides that, surface tension and contact angle under high pressure can be measured, to our knowledge, using the pendant drop method [17, 24–31] or the methods based on capillary waves [18] or capillary rise [16], which typically utilise visible light for the detection.

Recently, several advanced methods allowing for the observation of mass transport in bulk bodies have been reported. These methods involve the detection based on *i*) Raman spectroscopy, which was used to characterize diffusion in liquids and liquids at pressures near atmospheric [32, 33] and the distribution of methane in methane hydrate at pressures up to 140 bar [34], *ii*) dynamic light scattering, which was used for the studies of diffusion of gases in liquids at pressures up to 34 bar [35], *iii*) Nuclear Magnetic Resonance (NMR), which was used for the studies on diffusion in liquids at pressures close to atmospheric [36–38], propane diffusion in liquids at pressures up to 10 bar [39] or methane distribution in methane hydrate at pressures up to 250 bar [40], *iv*) X-ray tomography, which was used for the studies on diffusion in liquids at pressures near atmospheric [38, 41] and methane hydrate formation at pressures up to 62 bar [42], and, most recently, *v*) neutron imaging, which was used for the observation of ammonium absorption in solids at pressures near atmospheric [43].

When compared to other radiations and fields, neutrons not only allow for the highly spatially resolved radiography [44] but also show high penetrability through metals and high sensitivity against hydrogen [45]. Hence, neutron imaging appears rather powerful when compared to the other methods. To our knowledge, neutron imaging has not been previously used to study the absorption of gases in liquids or surface phenomena at high pressures. We did so as highly mechanically stable and well transparent measuring cells can be constructed, in the case of neutron imaging, from titanium or from other metals. To demonstrate the properties of the method, the *in-situ* measurement of the rather fast transient diffusion of methane into ethanol liquid bodies and their swelling at high pressures is reported. Besides that, neutron imaging routinely provides images having the spatial resolution in sub-100 μm domain [45] and recently in sub-10 μm domain [44], enabling the visualization of liquid menisci in metal vessels under high pressures.

Neutron rays are attenuated rather strongly in materials containing protium (^1^H), while compounds containing deuterium (^2^H or D) appear significantly more transparent. We have therefore measured the diffusion, liquid swelling and surface phenomena for the two pairs: methane (CH_4_) with perdeuterated ethanol (C_2_D_6_O) and methane (CH_4_) with perdeuterated *n*-decane (*n*-C_10_D_22_). Similar to the one-pot strategy widely applied in chemistry for conducting multiple chemical transformations in one chemical reactor [46–48], multiple characteristics were derived by our method from one probe within each one-pot experiment.

Although density of a liquid naturally depends on the isotopic composition of the compound, its molar volume typically does not vary significantly [49–51]. Besides that, surface tensions of liquids show minor dependences on their isotopic compositions [52] for a series of perdeuterated and perprotonated (normal) C_6_-C_8_ alkanes and aromatic compounds; the differences in the surface tensions of these liquids ranged 0.5–0.8 mN·m^−1^ or less, that is, approximately, 5% or less. By assuming the validity of the Wilke-Chang predictive correlation [53, 54], the mutual diffusivity, *D*, of methane in perprotonated ethanol can be expected to be by about 6% lower than that of methane (solute) in perdeuterated ethanol: by assuming that other parameters than molar masses, *M*, of the solvents (C_2_D_6_O and C_2_H_6_O) are equal, it follows that *D*_solute_ = const·(*M*_solvent_)^1/2^. Moreover, the isotopic fractionation due to diffusion in water shows, for instance, $D_{{}^{7}\text{Li}}/D_{{}^{6}\text{Li}}=0.99772$ ref. [55], or $D_{{}^{86}\text{Kr}}/D_{{}^{84}\text{Kr}}=0.9965$, ref. [56], thus suggesting that even smaller influence of the isotopical composition on diffusivity than that predicted based on the Wilke-Chang correlation can be expected. Thus, as limited differences of the physical properties occur due to the interchange of protium and deuterium in the studied molecules, the presented results are relevant not only for the deuterated but also for the protonated “normal” chemicals.

The experiments reported here were conducted with supercritical methane. However, density [57] and diffusivity of methane [10, 11], surface tension of the liquid exposed to methane [26] and equilibrium solubility [7, 9, 58, 59] of methane in liquids do not qualitatively change if pressure exceeds the critical pressure of methane within the conditions relevant to this work. The main reason for the use of supercritical methane (*p* > *p*_c_, *T* > *T*c) in this work was its higher density and, thus, higher neutronic contrast when compared to methane gas (*p* < *p*_c_, *T* > *T*c). Besides that, the supercritical conditions are also more relevant to the practical applications.

### Transient diffusion of methane in liquid bodies

One-dimensional transient diffusion of a gas (or supercritical fluid) into a liquid can be described with Fick’s law of diffusion as taken in the form of Equation 10.12 in the literature [60]:

$$\frac{\partial C_{A}^{B}}{\partial t}=D_{A}^{B}\frac{\partial^{2}C_{A}^{B}}{\partial \xi_{B}^{2}}$$ (1)

where $C_{A}^{B}$ is the concentration of compound A defined here as the number of moles of A per basic number of moles of B, that is, per the initial amount of pure compound B, *t* is time. The length coordinate ξ_B_ is defined so that unit ξ_B_ contains, per unit area, unit amount of compound B. This frame of reference implies that diffusion of A is measured with respect to compound B and $D_{B}^{B}=0$. Hence, if volume change occurs during diffusion in a flat sheet made from compound B having the initial length *L*^0^ and actual length *L*, it holds for the length coordinate ξ_B_ ∈ (0, *L*^0^), while for the overall length variable it holds *x* ∈ (0, *L*). In this work, A = CH_4_ and B = C_2_D_6_O or *n*-C_10_D_22_. For simplicity, following symbols will be used below: ξ = ξ_B_, $D=D_{{CH}_{4}}^{B}$ and $C_{A}=C_{CH4}=C_{{CH}_{4}}^{B}$. Clearly, following relations hold for molar fraction and molar concentration: *C*_A_ = *x*_A_/(1 − *x*_A_) and *C*_A_ = *c*_A_/*c*_B_. Please note that *x* without subscript refers to overall length variable while with subscript to molar fraction. Molar concentrations *c*_A_ and *c*_B_ have the usual meaning of the number of moles of the species per the total volume of the mixture.

The above choice of the reference frame is convenient as it coincides with the case in which swelling is negligible and constant thickness of the flat sheet equal to its initial thickness is assumed. This is the case of the classical total-uptake experiments during which the overall change of the length (or volume) of the absorbing phase is not known. The above choice of geometry (diffusion in one dimension), represents a simplification neglecting the real geometry of the meniscus. However, the influence of this simplification is minimized by measuring the concentration profiles in the centre of the cylinder and was tested by using different levels of the liquid (see below).

As a consequence of the above choice of the reference frame and geometry, Eq (1) can be solved analytically. One-dimensional diffusion of methane into cylindrical liquid bodies having the initial length (liquid level) *L*^0^ enclosed in impermeable walls from all sides other than from the top are studied below. Hence, the concentration profiles (analytical solutions of Eq (1)) have the form of the Equations 4.16, 4.17 and 4.29 in the literature [60], in which following substitutions were made: *l* = *L*^0^ and *x* = ξ_B_. Thus, the surfaces of the liquid body are located at -*L*^0^ and *L*^0^ and the central symmetry plane, which corresponds to the impermeable bottom wall, is located at zero; the profiles at negative length coordinates are hypothetical.

In the case of the uniform and negligibly small concentration distribution of A within the liquid body and instantaneous change of pressure of A and thus its boundary concentration at zero time, the concentration profile of A in the liquid B has the form:

$$C_{A}=C_{A}^{L^{0}}\left\{1-\frac{4}{\pi }\sum_{0}^{\infty }\frac{{\left(-1\right)}^{n}}{2n+1}\text{exp}\left[-\frac{D{\left(2n+1\right)}^{2}\pi^{2}t}{{\left(2L^{0}\right)}^{2}}\right]\text{cos}\frac{(2n+1)\pi \xi_{B}}{2L^{0}}\right\}$$ (2)

where $C_{A}^{L^{0}}$ is the methane concentration at the boundary (liquid surface). In this work, the initial concentration of the diffusing compound was approx. 1/80 or less of that at the boundary, see below, and was neglected.

In the same case as above but for a non-instantaneous initial change of the concentration of A at the boundary, the concentration profile of A in the liquid B has the form:

$$C_{\text{A}}=C_{\text{A}}^{L^{0}}\left\{1-\text{exp}(-\beta t)\frac{\text{cos}(\xi_{\text{B}}\sqrt{\frac{\beta }{D}})}{\text{cos}(L^{0}\sqrt{\frac{\beta }{D}})}-\frac{16\beta {\left(L^{0}\right)}^{2}}{\pi }\overset{\infty }{\underset{0}{\sum }}\frac{{\left(-1\right)}^{n}\text{exp}[-\frac{D{\left(2n+1\right)}^{2}\pi^{2}t}{{\left(2L^{0}\right)}^{2}}]}{\left(2n+1)\cdot [4\beta {\left(L^{0}\right)}^{2}-D\pi^{2}{\left(2n+1\right)}^{2}\right]}\text{cos}\frac{(2n+1)\pi \xi_{\text{B}}}{2L^{0}}\right\}$$ (3)

in which *β* has the meaning of the reciprocal time constant of the increase of the momentary boundary concentration, thus

$$C_{A,momenrary}^{L^{0}}=C_{A}^{L^{0}}[1-\text{exp}\left(-\beta t\right)]$$ (4)

In the case of the non-uniform initial distribution of the component A within the liquid B, which has, in this work, the form of Eq (2) or Eq (3), the concentration profile after instantaneous change of pressure and thus boundary concentration of A at zero time has the form

$$\begin{array}{l} C_{\text{A}}=C_{\text{A}}^{L^{0}}+\frac{2}{\pi }\sum_{1}^{\infty }\frac{C_{\text{A}}^{L^{0}}(\text{cos}n\pi -1)}{n}\text{sin}\frac{n\pi \xi_{\text{B}}}{2L^{0}}\text{exp}(-\frac{Dn^{2}\pi^{2}t}{2L^{0}})\\ +\frac{2}{2L^{0}}\sum_{1}^{\infty }\text{sin}\frac{n\pi \xi_{\text{B}}}{2L^{0}}\text{exp}(-\frac{Dn^{2}\pi^{2}t}{2L^{0}})\int_{-L^{0}}^{L^{0}}f(\xi_{\text{B}}^{\prime })\text{sin}\frac{n\pi \xi_{\text{B}}^{\prime }}{2L^{0}}\text{d}\xi_{\text{B}}^{\prime } \end{array}$$ (5)

where $f(\xi_{B}^{\prime })$ is the initial concentration distribution. In Eqs (2), (3) and (5), the use of low number (*e*.*g*. twenty) of the summation terms is sufficient due to the fast convergence of the sums.

### Swelling

Volume changes due to the diffusion of component A in a liquid body are generally negligible at high dilution. The molar volume, molar concentration and density of the liquid body composing of species A and B can be expressed using partial molar volumes [53] of the components and their amount in the solution:

$$V_{m}=1/c=(x_{A}M_{A}+x_{B}M_{B})/\rho ={x_{A}\overset{-}{V}}_{A}+{x_{B}\overset{-}{V}}_{B}$$ (6)

As *x*_A_ ≫ *x*_A_ in the studied systems, following simplifications are made below: partial molar volume of B is set here to be equal to the molar volume of the pure liquid B, ${\overset{-}{V}}_{B}=V_{m,B}^{0}$, and ${\overset{-}{V}}_{A}$ is assumed to be concentration independent.

The level of the liquid body having the shape of cylinder during the one-dimensional diffusion of compound A can thus be calculated based on the actual concentration profiles using Eq (6), thus:

$$L=L^{0}+\frac{{\overset{-}{V}}_{A}}{V_{m,B}^{0}}\int_{\xi_{B}=0}^{\xi_{B}=L^{0}}C_{A}d\xi_{B}$$ (7)

The models of diffusion and swelling, as described in sections 1.1 and 1.2, provide a rather classical description of diffusion and liquid swelling using purely analytical expressions, which is a viable alternative to the commonly employed methods based on the numerical modelling [39, 41].

### Shape of liquid meniscus in a tube

Based on the Young-Laplace equation, the shape of the liquid-gas (here liquid-supercritical fluid) interface in a tube of axial symmetry and non-negligible diameter follows [61, 62] the differential equation

$$z=\frac{\gamma }{\Delta \rho g}\left(\frac{z\prime \prime }{1+{z\prime }^{2}}+\frac{z\prime }{y{(1+{z\prime }^{2})}^{1/2}}\right)$$ (8)

in which *z* is the meniscus profile, *y* ∈ (0, *r*) spatial coordinate, *r* inner diameter of the tube, Δ*ρ* is the difference of the densities of the liquid and gaseous (supercritical fluid) phases, *g* = 9.80740 m·s^-2^ is gravity in Villigen [63], *γ* is surface tension. As Eq (8) can be numerically solved under conditions *z*′(*y* = 0) = 0 and *z*′(*y* = *r*) = cot *θ*, three adjustable parameters (contact angle *θ*, surface tension, position of the meniscus) can be calculated by fitting the calculated profiles to the experimental menisci shapes.

The dependence of the surface tension on pressure in a binary system methane-liquid follows [26]

$${\left(\frac{\partial \gamma }{\partial p}\right)}_{Area,T}=-c_{A}^{S}\frac{{(1-x}_{A}^{liq.}){\overset{-}{V}}^{gas}-{(1-x}_{A}^{gas}){\overset{-}{V}}^{liq.}}{x_{A}^{gas}-x_{A}^{liq.}}≅-c_{A}^{S}{\overset{-}{V}}^{gas}$$ (9)

in which $c_{A}^{S}$ stands for the surface excess concentration of methane. Following the convention taken from the literature, it is assumed that ${\overset{-}{V}}^{gas}$ can be approximated by the molar volume of (pure) methane and the methane phase is denoted as “gas” despite the same formula can be used for the case of the supercritical fluid [26]; the right-hand side approximation holds for the systems at moderate pressures and negligible evaporation of the liquid. Specifically, following inequalities justify well that approximation for partial pressures of methane relevant to this work: $x_{C2H6O}^{gas}<0.004$ at 0 °C [7] and $x_{C2H6O}^{gas}<0.005$ at 40 °C [58] and $x_{C10H22}^{gas}\leq 0.0014$ at 37.8 °C [9] and $x_{n-C8H18}^{gas}\leq 0.003$ below 50 °C [59].

The surface excess concentration of methane thus yields [26]

$$c_{A}^{S}=-\frac{p}{zRT}{\left(\frac{\partial \gamma }{\partial p}\right)}_{Area,T}$$ (10)

in which *z* is the gas compressibility factor.

To our knowledge, no theory exists for the pressure dependence of the contact angle in binary systems of gas-liquid, while correlation was reported for CO_2_-water-coal [27]. On the contrary, theoretical treatment is available for the pressure dependence of the surface tension in the systems of comprising of liquid and its vapour [64]. Temperature dependence of surface tension of pure components can be correlated with various models, such as the Eötvös model [65] or some of the expansions used for the heavy and normal water [66, 67]. Similar to that, the contact angle of pure components (*n*-alkanes) on solid surfaces (teflon) was found to be temperature dependent [68] and theory was proposed [69].

## Materials and methods

### Materials

Following chemicals were used as received from the supplier: methane (CH_4_, 4.5, Messer, CAS 74-82-8), nitrogen (N_2_, 4.0, Messer, CAS 7727-37-9), deuterated ethanol (C_2_D_6_O, Cambridge Isotope Laboratories, isotopic enrichment 99.5%, chemical purity ≥99%, CAS 1516-08-1), deuterated *n*-decane (*n*-C_10_D_22_, Cambridge Isotope Laboratories, isotopic enrichment 99.6%, chemical purity 97.3%, CAS 16416-29-8), acetone (C_3_H_6_O, Penta, p.a., CAS 67-64-1, used as a cleaner).

Density of compounds containing deuterium has rarely been reported in the available literature, while it naturally differs from that of compounds containing normal hydrogen (protium). Based on the available literature data, molar volumes of deuterated compounds typically differ by 2% for water [49, 50] and 0.1% for chloroform [51] at pressures and temperatures relevant for this study. It thus seems meaningful to assume that the molar volumes of C_2_D_6_O and *n*-C_10_D_22_ equals those of C_2_H_6_O and *n*-C_10_H_22_ to within ≈1%. Isothermal compressibility of the protium-containing compounds was taken from the literature [70, 71] and was assumed to be equal to that of the deuterium-containing compounds. The state behaviour of methane was calculated with the Peng-Robinson equation of state [53, 72]. Physical constants of the compounds used in this work are summarized in Table 1.

**Table 1:** Physical properties of the chemicals.

chemical	*M*, g·mol^-1^	ρ^7.0 °C, sat.^, g·cm^-3^	ρ^37.8 °C, sat.^, g·cm^-3^
C_2_D_6_O	52.11 [73]		
C_2_H_6_O	46.07 [74]	0.800 [75],	0.774 [75],
		0.802 [74]	0.774 [74]
*n*-C_10_D_22_	164.42 [73]		
*n*-C_10_H_22_	142.28 [74]	0.740 [75],	0.716 [75],
		0.741 [74]	0.718 [74]
chemical	*T*_c_, K	*p*_c_, bar	acentic factor, ω
CH_4_	190.564 [74]	45.99 [74]	0.0115478 [74]

### Experimental setup

The measuring *in-situ* cells were made from Titanium Grade 5 (Ti-6Al-4V). The thermostating jacket was made from duralmin EN AW 6060, parts of apparatus not exposed to the neutron beam, such as pipes, valves and connectors, were made from stainless steel. The interior of the measuring cells was rinsed with acetone and purged with nitrogen prior to their use. The cells were maintained at constant temperature using a water circulator (Julabo F12-MA), temperature was measured with a Pt100 thermometer (Greisinger GMH 3710), absolute pressure was measured with a pressure transducer (Omega PXM409-175BAV, Omega DP41-B control unit), atmospheric pressure was sensed with a barometer (Greisinger GDH 11A).

**Fig 1:**
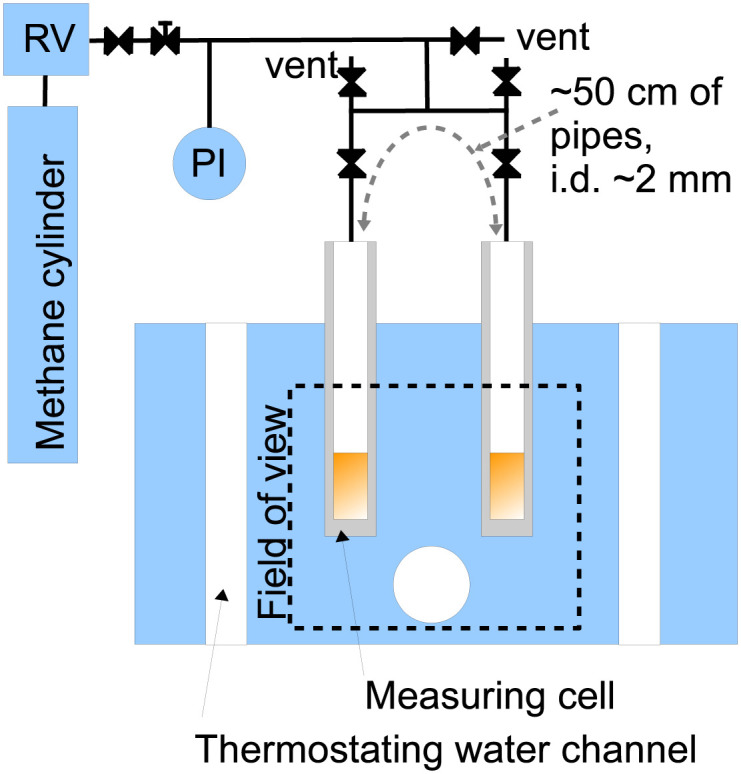
Principal scheme of the measuring apparatus. RV abbreviates reduction valve, PI pressure gauge with indication.

The apparatus consisted of two measuring cells placed in a duralmin body maintained at a constant temperature (Fig 1); the whole apparatus was placed in a 2 mm thick duralmin safety box continuously purged with nitrogen. Deuterated ethanol (C_2_D_6_O) was placed in one measuring cell (a cylinder with the centred 9.0±0.1 mm flat-bottomed bore and wall thickness 1.5 mm), deuterated *n*-decane (*n*-C_10_D_22_) in the second one; the typical liquid level in each cell was about 1–1.5 cm. The liquids were then separately bubbled for ~1 minute with nitrogen at a flow rate of ~20 cm^3^ min^-1^ using a stainless steel capillary connected to pressurized nitrogen temporarily submerged into the liquid. Afterwards, each liquid was bubbled for ~1 minute with methane at a flow rate of ~20 cm^3^ min^-1^ and the whole apparatus was purged with methane to remove other gases. The apparatus containing the liquid equilibrated with methane at atmospheric pressure was then gradually charged with the compressed methane; each pressure change took 15–30 seconds. During the experiments, the two cells remained connected to the reduction valve to assure constant pressure. These cells were thus connected, during the experiments, with approx. 50 cm of tubing having the inner diameter of approx. 2 mm, which effectively avoided potential contamination of the gaseous phases by free diffusion. After the experiment, each cell and the surrounding tubing was separately opened to the atmosphere and purged with the pure methane.

The experiments were performed at the measuring position no. 2 of the NEUTRA thermal neutron beamline at Swiss spallation Neutron source [76] (SINQ). The detailed beamline parameters are described elsewhere [45]. After traversing the sample, the incoming neutron beam was detected using MIDI-camera box fitted with 20-μm thick Gd_2_O_2_S/^6^LiF scintillator screen. The scintillation light has been collected using a 100-mm lens (Zeiss, Makro-Planar 2/100 ZF.2) onto a scientific complementary metal oxide semiconductor (sCMOS) camera detector (Hamamatsu ORCA Flash 4.0, pixel size 6.5 μm). This imaging arrangement provided images with the field of view of 43.2 × 43.2 mm × mm in size, in which the measuring cells and the thermostating jacket with the opening for the open beam were located, with the corresponding pixel size of 21.07 μm. The effective spatial resolution of the observations was assessed based on images of Siemens star resolution test object [77] to be about 50 μm (*i*.*e*. between 2 and 3 pixel sizes). This spatial resolution was vital for the observations of the changes of the liquid level, which typically yielded less than ~100 pixels, and, in particular, for the observation of the meniscus shape, the height of which was ~100 pixels and which was steep near the wall (see below). The practically negligible readout time of sCMOS detector allowed for continuous observation of the diffusion and liquid swelling processes. The exposure time of the single raw radiography was equal to 10 seconds. Apart from the dark current and open beam images that are standardly used for the quantification of neutron radiographies, the black body images (BB images) were acquired (as described in [78, 79]) to minimize the influence of the sample scattering and the background.

**Fig 2:**
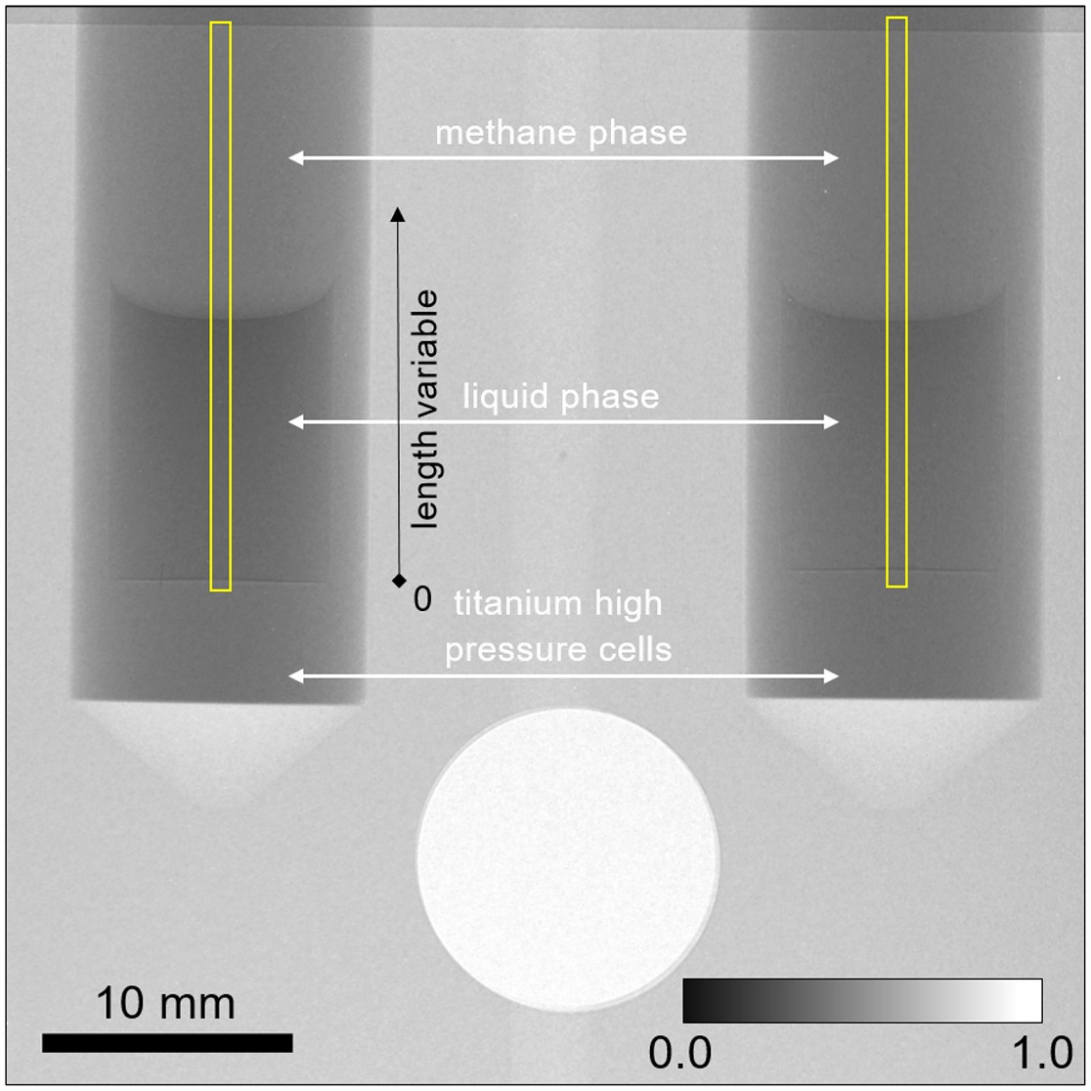
Neutron image of the setup 64.2 minutes after being pressurized with CH_4_ from 1.0 to 81.4 bar, 7.0 °C. Left cell: *n*-C_10_D_22_, right cell: C_2_D_6_O. Inner diameter of the measuring cell was 9.0±0.1 mm, outer 12 mm, grey intensity corresponds to transmittance. The regions used for the evaluation of the average intensity are depicted as yellow boxes; the methane phase and the length variable are indicated.

The sample attenuation was evaluated based on the neutron images of the empty cell and of the cell with the sample using Beer-Lambert law within the central part of the cell (Fig 2, yellow rectangle); the thicknesses of titanium and duralmin layers facing the neutron beam source equalled those facing the detector. The average intensity of the neutron rays was calculated for each length variable within the central part of the cell having the width of 40 pixels (0.868 mm; *i*.*e*. the width of the yellow box in Fig 2) and height corresponding to that of the interior of the measuring cell.

Beer-Lambert law for mixtures was used in the form

$$-\text{ln}\frac{\phi^{out}}{\phi^{in}}=A=\sigma_{A}N_{0}c_{A}d+\sigma_{B}N_{0}c_{B}d=\Sigma_{A}d+\Sigma_{B}d$$ (11)

where *A* stands for the apparent absorbance, which is referenced below as absorbance for simplicity, *σ* is cross section of the component, *N*_0_ = 6.02214076 ⋅ 10^23^ mol^−1^ is Avogadro constant [80], *d* stands for the path length and *c* for molar concentration (number of moles per unit volume), *ϕ*^in^ and *ϕ*^out^ are the intensities of the neutron irradiation entering and leaving the sample, *ϕ*^out^/*ϕ*^in^ is transmittance and *Σ* stands for the linear attenuation coefficient. The cross sections were evaluated by measuring the absorbance of the pressurized methane phase above the solution (Fig 2) and the absorbance of the liquid equilibrated with nitrogen under atmospheric pressure (1.0 bar): σ_C2D6O_ = (46±5) barn, σ_*n*−C2D6O_ = (173±9) barn, σ_CH4_ = (190±15) barn. The contributions of the neutron attenuation of the dissolved nitrogen and of the vapours of the studied compounds in the methane phase were neglected; see comments below Eq (9). The influence of the actual pressure on the concentrations was evaluated as described in Section 2.1. Averages of series of 50 radiographies were used for further evaluations. Time equal to one half of the duration of the series was assigned to the averaged neutron image.

## Results and discussion

A series of experiments on methane (CH_4_) absorption into still liquid bodies of deuterated ethanol (C_2_D_6_O) and *n*-decane (*n*-C_10_D_22_) was conducted at (7.0±0.5) °C and at (37.8±0.5) °C. These experiments were made in such a way that methane pressure was stepwise elevated from atmospheric pressure to approx. 80 bar and then to approx. 120 bar (see below for the measured pressures). Neutron radiographies were then acquired to follow diffusion and change in the level of the liquids (swelling). In the case of the experiments at (7.0±0.5) °C, methane pressure was stepwise changed from atmospheric pressure to approx. 120 bar for comparison.

**Fig 3:**
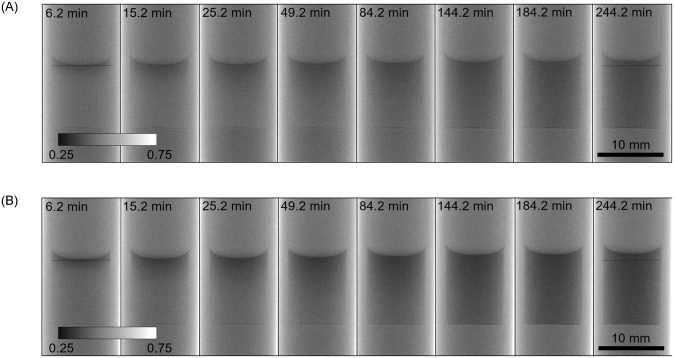
Series of neutron images of the cell initially filled with ethanol (C_2_D_6_O, A) and *n*-decane (*n*-C_10_D_22_, B) at 7.0 °C. Times elapsed after the change of methane pressure from 1.0 to 81.4 bar are indicated. Inner diameter of each cell (the diameter of the cylindrical liquid body) was 9.0±0.1 mm, grey intensity corresponds to transmittance. Processed data are shown in Figs 4 and 5.

The neutron imaging of the above described methane diffusion experiments provided dependences of absorbance of the liquid phase on the length variable and time. Space-resolved absorbance of the liquid body was clearly subject to changes, as well as the overall level of the liquid (Fig 3); more pronounced changes were observed for *n*-decane which shows higher methane solubility than ethanol. Hence, not only the concentration of CH_4_ but also that of the liquid became space- and time-dependent, thereby avoiding the assumption that absorbance of ethanol in each pixel remains constant. Hence, the general form of Eq (11) was used for the processing of the radiographies.

As the overall liquid level (length) depends linearly on the methane concentration, the overall liquid level was approximately expressed as

$$L=L^{0}(1+\sum a(A-A_{B}^{pure}))$$ (12)

where $A_{B}^{pure}$ is the absorbance of the pure liquid at the temperature and pressure of the system; summation was made over the entire liquid and *a* is a fitting parameter. Thus, the dilution of the component B with the component A was neglected for simplification. The contribution of methane to the overall absorbance, *A*_A_, was evaluated in each averaged pixel (defined above Eq (11)) of the image of the liquid body as

$$A_{A}=A-\frac{A_{B}^{pure}}{a(A-A_{B}^{pure})}=A-A_{B}$$ (13)

Concentrations of the individual components in each averaged pixel were then calculated based on the Beer-Lambert law, see Eq (6). The height of each pixel of the image of the liquid body was scaled by the factor $c_{B}/c_{B}^{pure}\leq 1$, thus obtaining the B-fixed length coordinate ξ_B_*ϵ*(0, *L*_0_) defined in Section 1.1. Overall, diffusion was parameterized with Eqs (2), (3) and (5) and swelling of the liquid with Eq (7).

### Diffusion of CH_4_ in the liquids and their swelling

Diffusion of methane in still liquid bodies was measured at 7.0 °C and 37.8 °C. In order to explore the effect of the initial level (length) of the liquid, two liquid levels (approx. 1.5 and 1.0 cm) and two modes of the methane pressure increase (1.0 → 118.7 bar and 1.0 → ≈82 → ≈118 bar) were realized. The observed concentration profiles were well parameterized with Eqs (2), (3) and (5) having the following adjustable parameters: diffusivity, *D*, concentration of methane at the boundary, $C_{A}^{0}$, and reciprocal time constant, *β* [Eq (4)]. Good agreement with the prediction of diffusivity at high dilution based on the Wilke-Chang correlation [53, 54] was found for *n*-decane and acceptable agreement for ethanol. This suggests that diffusivity was dependent on temperature in the sense of the Stokes-Einstein relation, based on which Wilke-Chang correlation was derived. Moreover, diffusivity observed for *n*-decane was well consistent with that from the literature [10]; no literature data are, to our knowledge, available for methane diffusivity in ethanol. Overall, the prediction can be considered to be less accurate than the measurement; see Tables 2 and 3 for the actual values. No significant influence of pressure on the diffusivity was observed within the experimental uncertainty.

**Table 2:** Parameters of Eqs (2), (3), (5) and (7) observed from our neutron imaging experiments on the diffusion of methane (CH_4_) in ethanol (C_2_D_6_O) and *n*-decane (*n*-C_10_D_22_) at 7.0 and 37.8 °C.

Methane (CH_4_) and ethanol (C_2_D_6_O)								
Temperature, °C	*p*_CH4_, bar	*x*_CH4_ at *p*_CH4_	10^9^·*D*, m^2^·s^-1^	1/*β*, s	ρ^D^, g·cm^-3^	ρ^H^, g·cm^-3^	${\overset{-}{V}}_{\text{CH}4}$, cm^3^·mol^-1^	Pressure change, bar
7.0	81.4	0.103^#^	2.9^#^	238^#β^	0.86^#^	0.76^#^	48^#^	1.0→81.4
		0.105^#β^	3.1^#β^		0.83^#β^	0.73^#β^	43^#β^	
		0.102^Ψ^			0.87^Ψ^	0.77^Ψ^	49^Ψ^	
7.0	117.7	0.137^##^	2.8^##^	*NA-low*	0.87^##^	0.77^##^	39^##^	81.4→117.7
		0.140^Ψ^			0.85^Ψ^	0.75^Ψ^	47^Ψ^	
7.0	118.7	0.119^#^	2.8^#^	183^#β^	0.85^#^	0.75^#^	53^#^	1.0→118.7
		0.120^#β^	2.9^#β^		0.78^#β^	0.70^#β^	50^#β^	
		0.137^Ψ^			0.85^Ψ^	0.76^Ψ^	45^Ψ^	
37.8	82.6	0.083^#^	4.9^#^	130^#β^	0.86^#^	0.77^#^	34^#^	1.0→82.6
		0.085^#β^	5.3^#β^		0.80^#β^	0.71^#β^	34^#β^	
		0.098^Ψ^			0.87^Ψ^	0.77^Ψ^	29^Ψ^	
37.8	118.7	0.111^#^	4.8^##^	*NA-low*	0.85^#^	0.75^#^	41^#^	82.6→118.7
		0.125^Ψ^			0.86^Ψ^	0.77^Ψ^	30^Ψ^	
Methane (CH_4_) and *n*-decane (*n*-C_10_D_22_)								
Temperature, °C	*p*_CH4_, bar	*x*_CH4_ at *p*_CH4_	10^9^·*D*, m^2^·s^-1^	1/*β*, s	ρ^D^, g·cm^-3^	ρ^H^, g·cm^-3^	${\overset{-}{V}}_{\text{CH}4}$, cm^3^·mol^-1^	Pressure change, bar
7.0	81.4	0.352^#^	3.0^#^	275^#β^	0.79^#^	0.69^#^	53^#^	1.0→81.4
		0.353^#β^	3.2^#β^		0.83^#β^	0.73^#β^	48^#β^	
		0.370^Ψ^			0.79^Ψ^	0.69^Ψ^	49^Ψ^	
					0.80^Ψ^^β^	0.70^Ψ^^β^	47^Ψ^^β^	
7.0	117.7	0.440^##^	2.9^##^	*NA-low*	0.78^##^	0.68^##^	48^##^	81.4→117.7
		0.465^Ψ^			0.77^Ψ^	0.67^Ψ^	48^Ψ^	
7.0	118.7	0.399^#^	3.1^#^	208^#β^	0.76^#^	0.66^#^	61^#^	1.0→118.7
		0.397^#β^	3.2^#β^		0.82^#β^	0.71^#β^	59^#β^	
		0.448^Ψ^			0.77^Ψ^	0.67^Ψ^	50^Ψ^	
37.8	82.6	0.306^#^	4.7^#^	176^#β^	0.78^#^	0.68^#^	51^#^	1.0→82.6
		0.317^#β^	5.4^#β^		0.80^#β^	0.70^#β^	43^#β^	
		0.348^Ψ^			0.79^Ψ^	0.69^Ψ^	42^Ψ^	
37.8	118.7	0.390^##^	5.0^##^	*NA-low*	0.80^##^	0.70^##^	37^##^	82.6→118.7
		0.428^Ψ^			0.78^Ψ^	0.68^Ψ^	42^Ψ^	

^#^ calculated by fitting Eq (2) or Eqs (2) and (7) to our experimental data

^#β^ calculated by fitting Eq (3) or Eqs (3) and (7) to our experimental data

^##^ calculated by fitting Eq (5) or Eqs (5) and (7) to our experimental data

^Ψ^ calculated from our data on the extrapolated liquid level and concentration, see Section 3.1.

**Table 3:** Parameters of Eqs (2), (5) and (7) derived from the literature data for the setting of the experiment.

Methane (CH_4_) and ethanol (C_2_D_6_O)						
Temperature, °C	*p*_CH4_, bar	*x*_CH4_ at *p*_CH4_	10^9^·*D*, m^2^·s^-1^	ρ^H^, g·cm^-3^	${\overset{-}{V}}_{\text{CH}4}$, cm^3^·mol^-1^	Pressure change, bar
7.0	81.4	0.100*[7], 0.092*[8], 0.096*^@^[6]	1.3^&^	0.76*^@^[6]	54*^@^[6]	1.0→81.4
7.0	117.7	0.137*[7], 0.130*[8], 0.129*^@^[6]	1.3^&^	0.74*^@^[6]	56*^@^[6]	81.4→117.7
7.0	118.7	0.138*[7], 0.131*[8], 0.130*^@^[6]	1.3^&^	0.74*^@^[6]	56*^@^[6]	1.0→118.7
37.8	82.6	0.084*[8]	2.7^&^			1.0→82.6
37.8	118.7	0.120*[8]	2.7^&^			82.6→118.7
Methane (CH_4_) and *n*-decane (*n*-C_10_D_22_)						
Temperature, °C	*p*_CH4_, bar	*x*_CH4_ at *p*_CH4_	10^9^·*D*, m^2^·s^-1^	${\overset{-}{V}}_{\text{CH}4}$, cm^3^·mol^-1^		Pressure change, bar
7.0	81.4	0.332*[15]	2.6^&^			1.0→81.4
7.0	117.7		2.6^&^			81.4→117.7
7.0	118.7		2.6^&^			1.0→118.7
37.8	82.6	0.315*[12]	5.5**[10]	59.6*****[14, 81]		1.0→82.6
		0.308#[83]	4.7^&^	55.4*****[13, 14]		
37.8	118.7	0.401#[83]	5.9***[10]			82.6→118.7
			8.5****[11]			
			4.7^&^			

* calculated from literature data for the temperature and pressure used in this work

*^@^ calculated from literature data for the pressure used in this work

** decane at 37.8 °C and 63.7 bar

***decane at 37.8 °C and 92.0 bar

**** dodecane at 45 °C and 100 bar

***** partial molar volume of methane in *n*-heptane at infinite dilution and at 25 °C

^&^ predicted using Wilke-Chang correlation [53, 54] with the parameters from the database [74] and molar masses from Table 1.

Since the concentration at the boundary corresponds to the locally equilibrated solution, equilibrium molar fractions of methane in the solutions were calculated (Table 2). The equilibrium solubilities compared well with the available literature data for ethanol [6–8] and *n*-decane [12, 15]. The influence of temperature on the equilibrium solubility and on the diffusivity followed the expected order. In all cases, the liquid level was well parameterized with Eq (7), thus allowing for the calculation of the partial molar volume of methane and the densities of the equilibrated solutions; fair agreement with the available literature data was observed [6, 13, 14, 81] (Table 2). Neither the mode of the pressurization (single step and two consecutive steps) nor the initial liquid level nor model equation, Eqs (2), (3) and (5), resulted to significantly different methane diffusivity, solubility and liquid density, thereby indicating good consistency of the method and its assumptions.

**Fig 4:**
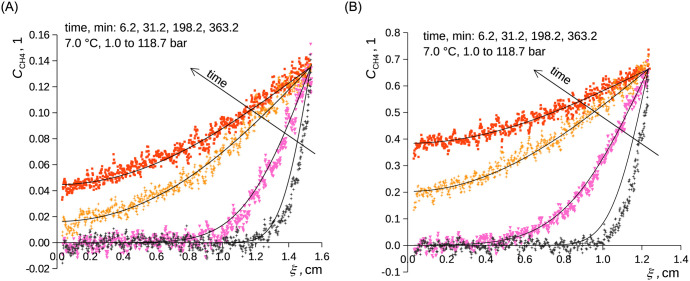
Relative concentration of CH_4_ in C_2_D_6_O (A) and in *n*-C_10_D_22_ (B) as a function of time and B-fixed length coordinate. Curves represent Eq (2), points represent measured data. Methane pressure was changed stepwise from 1.0 to 118.7 bar at zero time; 7.0 °C. A) ethanol. B) *n*-decane.

**Fig 5:**
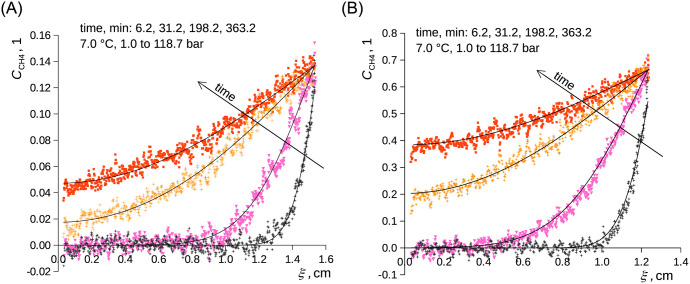
Relative concentration of CH_4_ in C_2_D_6_O (A) and in *n*-C_10_D_22_ (B) as a function of time and B-fixed length coordinate. Curves represent Eq (3), points represent measured data—the same experimental data as in Fig 4. Methane pressure was changed stepwise from 1.0 to 118.7 bar at zero time; 7.0 °C. A) ethanol. B) *n*-decane.

**Fig 6:**
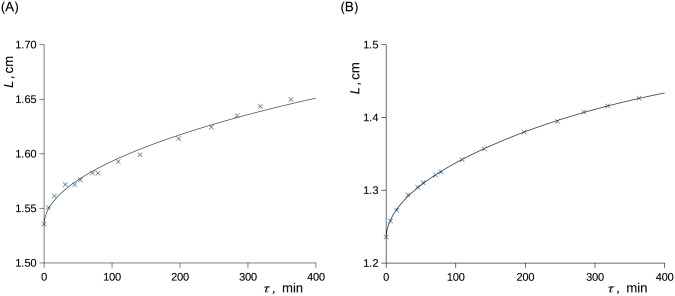
The overall liquid level during the absorption of CH_4_ in C_2_D_6_O (A) and in *n*-C_10_D_22_ (B). Curves represent Eq (7) with the use of Eq (2), points represent measured data. Methane pressure was changed stepwise from 1.0 to 118.7 bar at zero time; 7.0 °C. A) ethanol. B) *n*-decane.

For both studied liquids and both temperatures, a moderate but repeatable overestimation of the data by the model given by Eq (2) occurred at short times in the case of the experiments starting from the (low) atmospheric pressure and was more pronounced for *n*-decane than for ethanol (Fig 4); similar phenomena were earlier reported for the diffusion in diluted systems [38, 82]. The overestimation was easily observable within the spatial resolution of the device and was well parameterized for both investigated liquids using Eq (3) (Fig 5). By using Eq (3), we assume that the formation of the boundary layer shows inertia according to Eq (4) and thus slows the diffusion. This means that the boundary condition is not established immediately once the methane pressure increases while it follows Eq (4). Time constant of the build-up of the surface layer ranged 130–275 s and is reported in Table 2. Slower build-up was observed at the lower inspected values of pressure and temperature. Moreover, the time constant was by approx. 20% higher for *n*-decane than that for ethanol. The formation of the diffusion boundary is clearly rather complex as the initial stage of the experiment involves local temperature changes due to the release of the heats of pressurization and dissolution. We assume that these effects disturbed the formation of the layer of methane adsorbed on the liquid surface (see below for the actual surface excess values). The use of Eq (3) instead of Eq (2) had naturally almost no influence on the fit of the concentration profiles at longer times. The use of the simpler model of diffusion, Eq (2), was well sufficient for the parameterization of the liquid level using (Fig 6).

**Fig 7:**
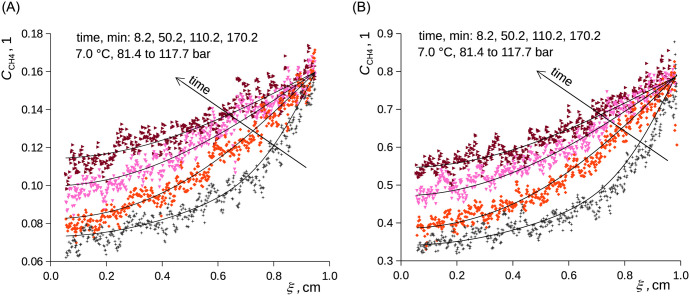
Relative concentration of CH_4_ in C_2_D_6_O (A) and in *n*-C_10_D_22_ (B) as a function of time and B-fixed length coordinate. Curves represent Eq (5), points represent measured data. Change of methane pressure from 81.4 to 117.7 bar, 7.0 °C. Pressure change (time zero) was made at time 254 minutes from the beginning of the 1.0 to 81.4 bar experiment. A) ethanol. B) *n*-decane.

Contrary to the experiments starting at low pressures, no significant overestimation of the first profiles with respect to Eq (5) was discerned in the case of the experiments starting at high pressure: ≈82 → ≈118 bar (Fig 7). Hence, the phenomenon slowing the progress of diffusion in the low pressure (concentration) regime, which we assume to have the nature of slow adsorption of methane onto the liquid surface, did not impose substantial resistance against diffusion at higher pressures as the layer of methane adsorbed on the interface was already formed before the pressure change and as lower local evolution of heat due to compression and dissolution occurred.

Alongside the above interpretation, several phenomena could potentially influence the course of diffusion and cannot be easily corrected, such as *i*) the approximate nature of Eqs (12) and (13) in combination with the steep concentration profile at low times *ii*) slow charging of methane to the sample (this took 15–30 seconds), *iii*) the above discussed local evolution of the heat of methane dissolution, methane compression and of the Joule-Thompson effect, thus influencing the boundary concentration at small times, *iv*) imperfection of the geometry at the liquid surface: flat surface is assumed in Eqs (2), (3) and (5), *v*) evaporation of the liquid, *vi*) limited mass transfer from the methane phase to the liquid phase due to the traces of impurities (nitrogen). As the concentration profiles were well parameterized in the case of pressure steps ≈82 → ≈118 bar without assuming any inertia at the boundary, the imperfection of the liquid surface geometry had little effect.

Since the above described evaluation of the equilibrium concentration of methane in C_2_D_6_O is based on the assumed validity of Eqs (2), (3) and (5) and on the approximation of the concentration of either C_2_D_6_O or *n*-C_10_D_22_ within the liquid body according to Eqs (12) and (13), the equilibrium concentrations were calculated by the following alternative method for comparison: The averaged absorbance of the liquid body and of the methane phase (Fig 2) was plotted against the length variable and the absorbance of the liquid at the interface was estimated by linear extrapolation, which corresponds to that of the equilibrated solution. Hence, the concentration of methane in the liquid was calculated based on Eq (11), in which the concentration of either C_2_D_6_O or *n*-C_10_D_22_ was estimated based on the equilibrium level of the liquid, obtained as the limit of Eq (7) at infinite time, and on the initial concentration of the liquid at the experimental pressure: $c_{B}^{\infty }=c_{B}^{0}L^{0}/L^{\infty }$. The equilibrium methane concentrations evaluated with this method showed, in most cases, better agreement with the literature data than the ones calculated by fitting Eqs (2), (3) and (5). The experimental (our) data are shown in Table 2, the available literature data are in Table 3.

### Surface tension and contact angle

**Fig 8:**
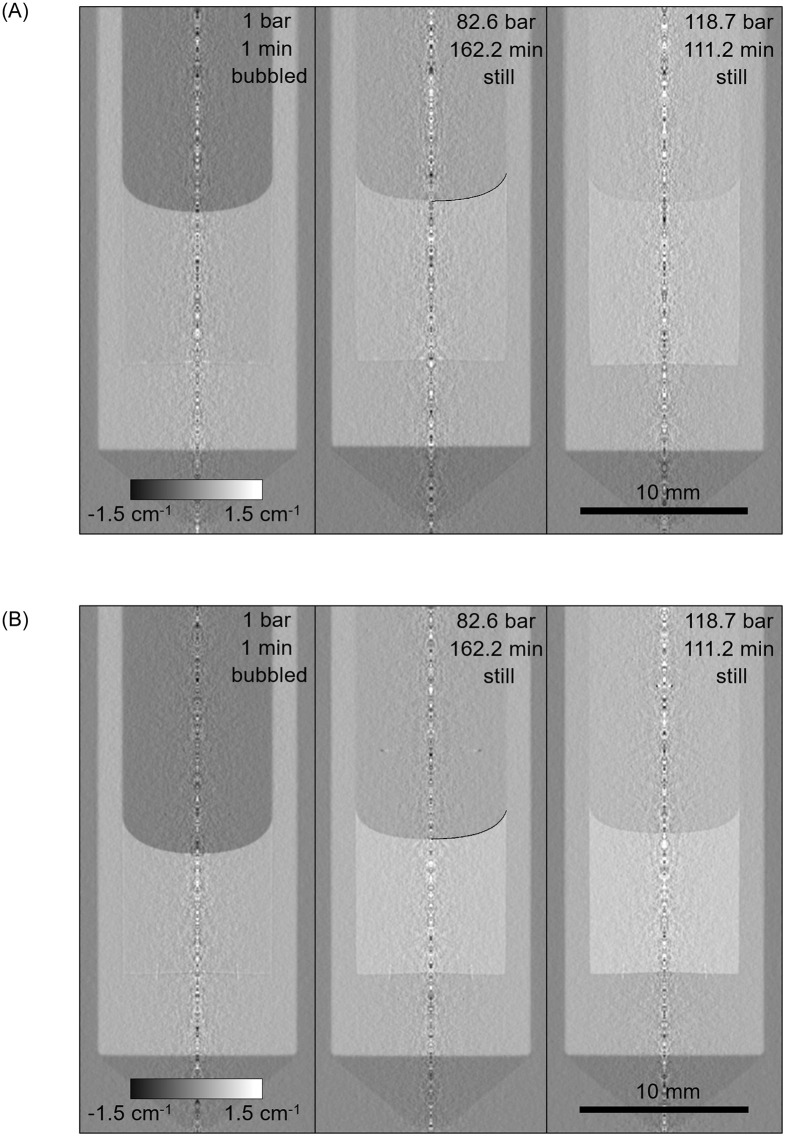
Tomographic reconstructions of the titanium cell high pressure with the liquid body and methane phase for 1 bar (left), 82.6 bar (middle) and 118.7 bar (right) in each triad of the reconstructions. The model of the meniscus, solution of Eq (8), is depicted as the black curve (at 82.6 bar, right half of the meniscus). Systems of C_2_D_6_O (A) and *n*-C_10_D_22_ (B) with CH_4_ at 37.8 °C are shown, grey intensity corresponds to the neutronic attenuation coefficient, *Σ*. The inner diameter of each measuring cell is 9.0±0.1 mm. A) ethanol. B) *n*-decane.

The meniscus shape was evaluated for each neutron radiography collected for the state closest to the absorption equilibrium, that is, at the longest equilibration time at which neutron radiographies were taken. Although true equilibrium was not reached, the concentration gradient near the phase interface was rather small at long times (Figs 4, 5 and 7) and we thus assume that this did not significantly disturb the methane adsorption on the interface. Based on the knowledge that the sample is axially symmetric, the transmission image measured by neutron radiography was converted to a tomographic slice using a method based on the onion peeling algorithm [84], in which the full geometrical description of the forward projection was used instead of its linear approximation. While the neutron radiographies are sufficient for the assessment of the concertation profile in the bulk liquid and its level (see section 3.1), the thus-obtained tomographic slices (Fig 8) are superior for the fitting of the model to the actual meniscus profile. Naturally, the linear attenuation coefficient of the methane phase as well as of the liquid phase increased with the methane pressure. The titanium high-presusure cell served here as a convenient reference material. The observed cross-section of Titanium Grade 5 (having the average mole-based ratios of the main constituents Ti:Al:V = 0.859:0.105:0.036 [85]) is σ_TiG5_ = (10.2±0.9) barn, *i*.*e*. *Σ*_TiG5_ = (0.57±0.05) cm^-1^, while the theoretical value for the alloy of this composition from NIST [86] is *Σ*_TiG5_ = 0.558 cm^-1^. The shape of the meniscus was approximated with the numerical solution of Eq (8), in which the parameters were calculated fitting the model to the experimental profile (Fig 8 and Table 4). Density difference, a parameter of Eq (8), was calculated based on ρ^D^ averaged for the all types of evaluation (#, #*β*, ## and Ψ), see Table 2, and on the state behaviour of pure methane as described with the Peng-Robinson equation of state [53, 72]. The evaporation of ethanol and *n*-decane into methane was neglected; see comments below Eq (9). The expected uncertainties of *θ* (±2 °) and *γ* (±2 mN·m^-1^) were estimated as the propagated uncertainties of how the numerical solution of Eq (8) fits the tomographic reconstructions of the menisci, the uncertainty of the diameter of the measuring cell and of the density difference. Similar to what was observed in section 3.1, no influence of the mode of the pressurization (single step and two consecutive steps) or of the initial liquid level (approx. 1 cm and 1.5 cm, both at 7.0 °C) on the measured quantities was discerned, thereby again indicating good consistency of the method.

**Table 4:** Contact angle and surface tension of liquid ethanol (C_2_D_6_O) saturated with methane (CH_4_) at given pressures and temperatures at Titanium Grade 5.

Methane (CH_4_) and ethanol (C_2_D_6_O)				
Temperature, °C	Pressure of CH_4_, bar	*γ*, mN·m^-1^	*θ*, °	Equilibration time, conditions
7.0	1.0	22, 24*	15	1 min, bubbled before exp.
7.0	81.4 (1.0→81.4)	15	14	244.2 min, still
7.0	117.7 (81.4→117.7)	12	14	170.2 min, still
7.0	1.0	22, 24*	15	1 min, bubbled before exp.
7.0	118.7 (1.0→118.7)	12	13	363.2 min, still
37.8	1.0	21, 21*	16	1 min, bubbled before exp.
37.8	82.6 (1.0→82.6)	14	14	162.2 min, still
37.8	118.7 (82.6→118.7)	11	13	111.2 min, still
Methane (CH_4_) and *n*-decane (*n*-C_10_D_22_)				
Temperature, °C	Pressure of CH_4_, bar	*γ*, mN·m^-1^	*θ*, °	Equilibration time, conditions
7.0	1.0	25, 25.1*	9	1 min, bubbled before exp.
7.0	81.4 (1.0→81.4)	15	8	244.2 min, still
7.0	117.7 (81.4→117.7)	11	10	170.2 min, still
7.0	1.0	25, 25.1*	8	1 min, bubbled before exp.
7.0	118.7 (1.0→118.7)	10	10	363.2 min, still
37.8	1.0	22, 22.2*	8	1 min, bubbled before exp.
37.8	82.6 (1.0→82.6)	13	12	162.2 min, still
37.8	118.7 (82.6→118.7)	10	11	111.2 min, still

* Data from the database [74] for ethanol (C_2_H_6_O) and *n*-decane (*n*-C_10_D_22_).

**Fig 9:**
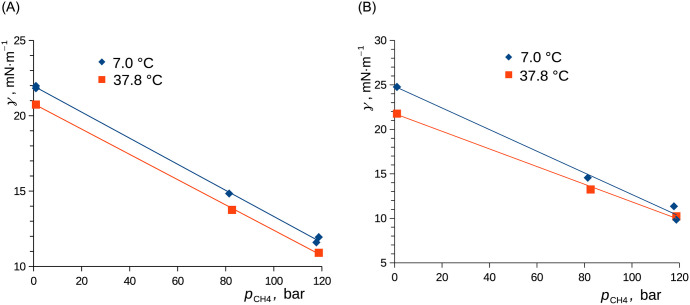
Surface tension of solutions of CH_4_ in C_2_D_6_O (A) and in *n*-C_10_D_22_ (B) as functions of methane (CH_4_) pressure. Linear fits calculated using the least squares method are shown. A) ethanol. B) *n*-decane.

Contact angle was found to be independent of temperature and methane pressure for both studied compounds within the inspected conditions and to within the experimental uncertainty. Surface tensions of pure perdeuterated ethanol and *n*-decane (neglecting dissolution of methane at 1.0 bar) were consistent with the available literature data (Table 4) and showed an expectable temperature dependence. The dependence of the surface tension on the methane pressure was well parameterized with the approximate form of Eq (9), see Fig 9. The pressure derivative yielded: (*∂γ*/*∂p*)_Area,7.0°C_ = −0.87 nm and (*∂γ*/*∂p*)_Area,37.8°C_ = −0.84 nm for ethanol and (*∂γ*/*∂p*)_Area,7.0°C_ = −1.22 nm and (*∂γ*/*∂p*)_Area,37.8°C_ = −0.99 nm = −0.99 · 10^−9^ · m^−1^ · Pa^−1^ for *n*-decane. The latter compares well with the value of around– 1.3 nm as calculated from the data reported for *n*-hexane and methane at 25 °C observed using the capillary rise method [16], for which the authors admitted that equilibrium was probably not reached, or– 0.88 nm reported for heptane in argon at 288 K as determined using the measurement of capillary waves [18]. Interestingly, the above derivatives are comparable to those reported in the literature [25, 26, 30] for water and methane (– 1.3 nm) despite the much lower methane solubility. The surface excess concentrations of methane on the surface of ethanol at 120 bar were $c_{A,7.0{}^{\circ}C}^{S}=5.8∙10^{-6}$ mol·m^-2^ and $c_{A,37.8{}^{\circ}C}^{S}=4.6∙10^{-6}$ mol·m^-2^, while the same quantity for *n*-decane yielded $c_{A,7.0{}^{\circ}C}^{S}=8.2∙10^{-6}$ mol·m^-2^ and $c_{A,37.8{}^{\circ}C}^{S}=5.5∙10^{-6}$ mol·m^-2^; see Eq (10). This compares well with the surface excess of (10 ± 2) · 10^−6^ mol·m^-2^ for the monolayer formed by spherically shaped methane molecules [26]. By seeing this together with the above-described inertia of the boundary concentration, we conclude that the interfacial layer of adsorbed methane formed rather slowly, that is, with the time constant of 130–275 s for the studied compounds and under the conditions inspected (Table 2).

**Fig 10:**
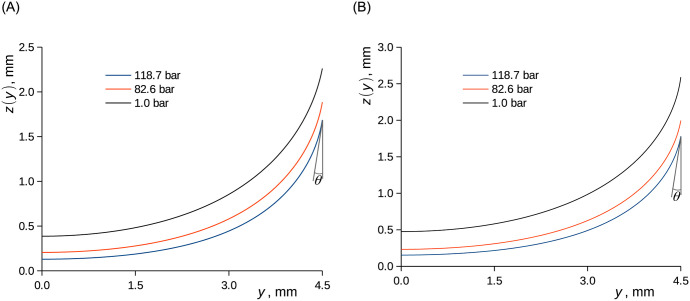
Menisci of solutions of CH_4_ in C_2_D_6_O (A) and in *n*-C_10_D_22_ (B) in a Titanium tube with the inner diameter of 9.0 mm at 37.8 °C. Curves represent the solution of Eq (8). Contact angle is schematically depicted (left). A) ethanol. B) *n*-decane.

**Fig 11:**
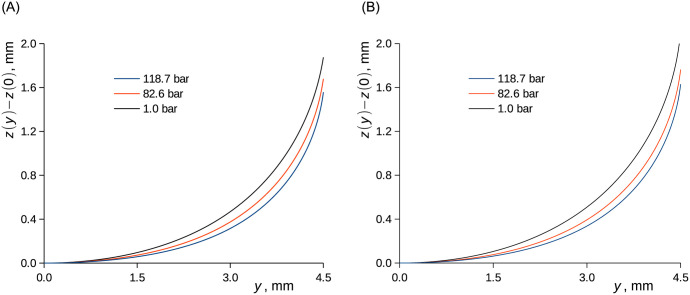
Menisci of solutions of CH_4_ in C_2_D_6_O (A) and in *n*-C_10_D_22_ (B) in a Titanium tube with the inner diameter of 9.0 mm at 37.8 °C. Curves represent the solution of Eq (8) from which the capillary elevation was subtracted. A) ethanol. B) *n*-decane.

The variation of the surface tension and, to a limited extent, contact angle with the methane pressure implies the changes of the capillary rise and meniscus shape (Figs 10 and 11). Clearly, the increase of methane pressure caused a drop of capillary elevation and a change of the curvature of the meniscus. The apparent surface tension of the liquid and its contact angle at the surface of the cell can be generally influenced by changes of adsorption of the components from the gaseous and liquid phase, which change their composition during the experiments [62]. The evaluation of such effects is, in principle, possible but experimentally challenging. We thus ascribe the changes to the changes of the surface energy due to methane adsorption, while the equilibrium adsorption on the solid surface can be well assumed.

Overall, this pioneering investigation not only extends the limits of the available methods, but also serves as a validation of the applicability of the neutron imaging technique for the phenomena having known basic principles. Clearly, this method can contribute in future to the explanation of not well understood phenomena occurring under harsh conditions. We prospect this method to be used to follow the kinetics of the methane hydrate formation and decomposition, which remains not completely understood [5, 87, 88], in particular for still liquids. These phenomena have consequences not only for the production, refining and transportation of the natural gas and crude oil, but also for the understanding of the methane release from the methane hydrate beds to the atmosphere due to climate changes.

## Conclusions

We report a new powerful method for capturing the time-resolved concentration profiles, liquid swelling and surface phenomena during the absorption of methane (CH_4_) in two still liquids (ethanol, C_2_D_6_O and *n*-decane, *n*-C_10_D_22_) in a one-pot experiment. The method, which is based on the neutron imaging of a cell containing a deuterated liquid upon its pressurization with a gas containing protium in its molecule, proved the ability to provide information on the methane diffusion into liquid, its swelling and capillarity from the single experiment in one pot. Hence, our new method enabled to collectively observe quantities, which could otherwise be studied only using multiple single-purpose methods, such as those for the visualization and measurement of diffusion in liquids [10, 11, 18, 21, 22, 32, 33, 35–39, 41], measurement of capillarity [16–18, 24–31] and liquid density [7, 8, 23]. Our method provided values of methane diffusivity and partial molar volume, liquid density of the solution, surface tension and its pressure derivative and contact angle at titanium under harsh conditions, while the observed quantities showed good agreement with the literature data where literature data or methods for their prediction are available [6–8, 10, 11, 13, 14, 16, 25, 26, 53, 54, 74, 81]. More so, the simultaneous observation of these phenomena with the pixel resolution of approx. 21 μm enabled, for the first time, to detect that the diffusion of methane in the liquid became anomalously slow in its initial stage upon the pressure change from atmospheric to 80 and to 120 bar; time constant of the slow boundary layer formation varied 130–275 s depending on the investigated component, methane pressure and temperature. Contrary to that, regular methane diffusion was observed for the methane pressure steps from 80 to 120 bar; the boundary layer formed instantaneously in this case in which a significant adsorption layer was already formed before the pressure step. Methane adsorption on the phase interface was thus found to be the likely limiting phenomenon for the methane diffusion into the liquid ethanol.

Clearly, neutron imaging represents a powerful tool for the simultaneous observation of gas dissolution and surface phenomena and can, in future, be used for studies of dissolution of methane or other hydrogen bearing gases in liquids relevant for the oil and gas production and for the observation of surface and diffusion phenomena in systems showing phase changes, such as for the formation and decomposition of methane hydrate.
